# DFSNet: A 3D Point Cloud Segmentation Network toward Trees Detection in an Orchard Scene

**DOI:** 10.3390/s24072244

**Published:** 2024-03-31

**Authors:** Xinrong Bu, Chao Liu, Hui Liu, Guanxue Yang, Yue Shen, Jie Xu

**Affiliations:** School of Electrical and Information Engineering, Jiangsu University, Zhenjiang 212000, China; 2212107002@stmail.ujs.edu.cn (X.B.); lc96222@163.com (C.L.); 2112107014@stmail.ujs.edu.cn (J.X.)

**Keywords:** semantic segmentation, point clouds, deep learning, tree protection robot

## Abstract

In order to guide orchard management robots to realize some tasks in orchard production such as autonomic navigation and precision spraying, this research proposed a deep-learning network called dynamic fusion segmentation network (DFSNet). The network contains a local feature aggregation (LFA) layer and a dynamic fusion segmentation architecture. The LFA layer uses the positional encoders for initial transforming embedding, and progressively aggregates local patterns via the multi-stage hierarchy. The fusion segmentation module (Fus-Seg) can format point tags by learning a multi-embedding space, and the generated tags can further mine the point cloud features. At the experimental stage, significant segmentation results of the DFSNet were demonstrated on the dataset of orchard fields, achieving an accuracy rate of 89.43% and an mIoU rate of 74.05%. DFSNet outperforms other semantic segmentation networks, such as PointNet, PointNet++, D-PointNet++, DGCNN, and Point-NN, with improved accuracies over them by 11.73%, 3.76%, 2.36%, and 2.74%, respectively, and improved mIoUs over the these networks by 28.19%, 9.89%, 6.33%, 9.89, and 24.69%, respectively, on the all-scale dataset (simple-scale dataset + complex-scale dataset). The proposed DFSNet can capture more information from orchard scene point clouds and provide more accurate point cloud segmentation results, which are beneficial to the management of orchards.

## 1. Introduction

In recent years, in the context of the modern agricultural industrial base and the rapid development of smart agriculture, the orchard industry has created substantail economic value, and it has become more prominent in agriculture [1,2,3]. However, with their limitations in perception capacity, some orchard management machines still rely on manual evaluation of tree growth and management, which is labor intensive and time consuming [4]. To ensure high-quality production in orchards, the implementation of perceptive technology is necessary for monitoring environments at any time for the efficient management of orchards. The unmanned orchard management robot, with some automatic functions such as object detecting, tree planting, protecting, variable spraying, fruit picking, and other tasks, has been widely used in the productive process of agroforestry [5,6]. The high-precision semantic segmentation technology of unstructured scenes is a key technology for orchard management robots, which need to understand the surrounding environment to achieve positioning and autonomous navigation [7]. The point cloud has increasingly been used in visual perception tasks of orchard management robotics [8], with its insensitivity to variances in lighting, shadows, and other factors. Point cloud-level semantic segmentation helps visual perception systems realize object recognition and detection, which greatly improves the operation and production efficiency of robots [9].

In recent years, deep learning technology has widely been used in 3D point clouds segmentation in agricultural scenes [10,11]. Chen Y et al. (2021) [7] replaced random sampling with farthest point sampling (FPS) to build local feature aggregation based on RandLa-Net. Furthermore, the RoseSegNet [12] was designed to identify phenotypic traits of roses in garden. RoseSegNet segmented plants into their structural components such as flowers, stems, and leaves. Although there are many studies for scenes segmentation in forestry and agriculture, the accuracy and applicability of scene segmentation are still challenging, such as the problems of point cloud in-homogeneity, sparsity, and permutation invariance [13,14]. Considering these problems, to enhance the ability of point clouds segmentation, the prevailing trend has been to add advanced local operators and create new structures of networks. The local feature aggregation (LFA) module plays a role in the network to build connections between centroids in point clouds. For example, some methods regard regular spatial kernels for local pattern encoding [15,16,17,18,19], and a series of methods utilize local geometry through edges [20,21,22]. Especially since PointNet++ [15] and D-PointNet++ [16] use farthest point sampling (FPS) to aggregate the local features of point clouds, the prevailing trend has been to add local feature aggregation modules to extract local features. FPS can ensure an even distribution of centroids within the point cloud and aggregate local features in the point cloud. DGCNN [20] constructs a local neighborhood graph by exploiting k-nearest neighbor (k-NN), and the updates of dynamic graphs make the receptive field as large as the diameter of the point cloud. PointMLP [19] uses a geometric affine module that can extract the local points before and after aggregation operations. In addition, the architecture of a network is also a necessary part that can be used to mine the features of point clouds efficiently. PointNet [23] and DGCNN use a single branch structure to make the network deeper, and concatenate global and local features to output per point scores. PointMLP uses a deep residual MLP network for point cloud analysis. PointNet++ uses a hierarchical feature learning architecture, and uses skip link concatenations to integrate features between layers. D-PointNet++ combines the architectures of DenseNet and PointNet++ to improve segmentation accuracy. Moreover, the emergence of transformer-based architectures [24,25,26] exhibited great success in vision tasks driven by a new spatial modeling mechanism based on dot product self-attention. To further utilize spatial characteristics of features in higher-order interactions, HorNet uses a recursive structure called g^n^Conv [27]. Although the various networks proposed in the above studies have a good effect on point cloud semantic segmentation, they do not fully consider the relationships between different layers in the network. The systematic comparison among representative methods is shown in Table 1.

In this research, an advanced 3D point cloud semantic segmentation neural network dynamic fusion segmentation network (DFSNet) for orchard scenes is proposed. The network leverages the local feature aggregation (LFA) module, and presents a fusion segmentation (Fus-Seg) architecture that fuses the segmental vectors from different layers. The LFA module was designed by Zhang R et al. (2023) [28], which includes sampling and grouping layers, trigonometric functions, and pooling operations. The module produces a high-dimensional local vector for point clouds with raw-point embedding and multi-stage hierarchy. The encoder conducts initial embedding to transform the raw coordinates of a point cloud into high-dimensional vectors, and progressively aggregates local patterns via the multi-stage hierarchy. The dynamic Fus-Seg architecture fuses two layers of the network, and generates feature tags of points by a learning multi-embedding space. This study introduces the construction process of the network in terms of deep mining of point clouds in detail. The experimental results show that the accuracy the neural network can be significantly improved by efficiently designing the fusion behavior of the different layers. To further promote the performance of the presented network, we discuss the impact of the combination of the different sampling and grouping strategies on LFA in point clouds. The primary contributions of this research are the following:

- A deep learning network that can perform semantic segmentation on 3D point cloud data in agricultural scenes.
- A concise but efficient network architecture that can fuse features from different layers in the network.
- The effect of different sampling strategies of a local feature aggregation module is discussed.
- Demonstrations of the proposed DFSNet, data labeling, and network training and prediction, providing end-to-end implementation for semantic segmentation in natural orchard fields.

## 2. Materials and Methods

The network is implemented in three phases: (1) The local feature aggregation module obtains local features of points; (2) the fusion segmentation module fuses different layers of the network and produces the class label of each point; (3) a dynamic network architecture is established, which is beneficial to recompute class labels using fusion segmentation modules.

### 2.1. Local Feature Aggregation Module

**Figure 1:**
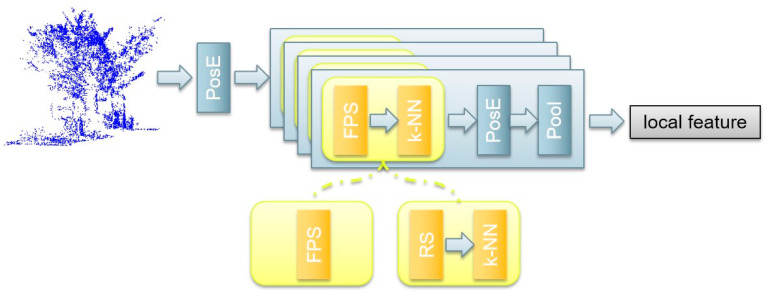
The local feature aggregation (LFA) module. Yellow blocks indicate the sampling and grouping strategies. PosE: positional encoder, FPS: farthest point sampling, RS: random sampling, k-NN: k-nearest neighbor grouping, Pool: max pooling. An SA (set abstraction) module includes a sampling and grouping strategies block, a PosE, and a Pool, and there are 4 stages of SA modules in the LFA.

The local feature aggregation (LFA) module combines the positional encoding in the transformer [29] with sampling and grouping strategies, which is shown in Figure 1. The positional encoder (PosE) transforms the raw coordinates $p_{i}=(x_{i},\;y_{i},\;z_{i})$ of the point cloud into high dimensional vectors to achieve feature embedding without learnable maps. The PosE utilizes the trigonometric function to encode the low feature vector of the point into a high dimensional vector during the inherent nature of trigonometric functions. The PosE can well capture fine-grained structural variations of 3D shapes and well encode relative positional information between different points in point clouds. Taking the encoding feature of point i on the x axis as an example, the process can be represented by the following formula:

(1) $$\begin{array}{c} f_{i}^{x}[2t]=\sin(\mu x_{i}/\nu^{\frac{6t}{F_{I}}})\\ f_{i}^{x}[2t+1]=\cos(\mu x_{i}/\nu^{\frac{6t}{F_{I}}}) \end{array}$$

where $f_{i}^{x}$ denotes the encoded feature vector on the x axis, $t\in [0,\;\frac{F_{I}}{6}]$ denotes the channel index, $F_{I}$ denotes the dimension of encoded feature vectors, and μ, ν control the magnitude and wavelengths, respectively. The special process of encoding the point coordinates into a higher dimensional vector is represented by Formula (2).

(2) $$\mathrm{PosE}(p_{i})=Concat(f_{i}^{x},\;f_{i}^{y},\;f_{i}^{z})\in R^{1\times F_{i}}$$

where $f_{i}^{x},\;f_{i}^{y},\;f_{i}^{z}\;\in R^{1\times \frac{F_{I}}{3}}$ denote the encoded vectors on three axes.

The other core of the local feature aggregation module is the set abstraction (SA) module. The iterative SA layer can process the point cloud in each hierarchical level and perform dense prediction by propagating point features along the neighbor region. The SA layer comprises the sampling layer, the positional encoding, and the pooling. The sampling layer is used to find the neighbor points within a local region of each centroid. Different methods can be used to obtain the centroid, including farthest point sampling (FPS) and random sampling (RS). After that, the k-NN is used to group neighbor points among the centroid. The PosE can reveal the local patterns, and the pooling layer utilizes both max and average pooling for local feature aggregation. In each SA layer, we obtain the local aggregated centers, which are fed into the next stage SA layer. Finally, after all 4 stages, a local feature map of the input point cloud is acquired.

### 2.2. Fusion Segmentation Module

**Figure 2:**
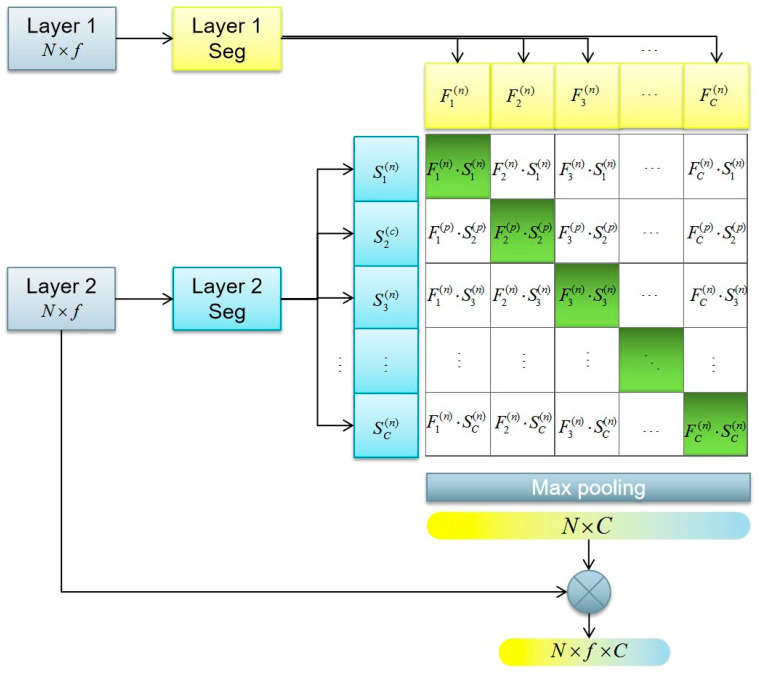
Fusion segmentation module (Fus-Seg). While standard convolution jointly trains point cloud feature segmentation (Seg) to predict point labels, the multi-model embedding space jointly trains two layers’ segmentation to predict the correct pairings of a batch of (Layer 1 and Layer 2) training examples. $\left\{F_{1}^{\left(n\right)},\;F_{2}^{\left(n\right)},\;F_{3}^{\left(n\right)},\;\cdots ,\;F_{C}^{\left(n\right)}\right\}$: the pre-labels of one point in a point cloud are obtained by Layer 1 Seg; $\left\{S_{1}^{\left(n\right)},\;S_{2}^{\left(n\right)},\;S_{3}^{\left(n\right)},\;\cdots ,\;S_{C}^{\left(n\right)}\right\}$: the pre-labels of the same point are obtained by Layer 2 Seg. N×f: feature map size of input point clouds; N×f×C: the feature map size of output point clouds; C: the segmentation number of DFSNet.

In Figure 2, the fusion segmentation module (Fus-Seg) shows an intuitive synergy [30,31] between different layers in the network. Then, the module generates labels for every point in the point cloud to extend the expression dimensions of the point cloud features.

When inputting a batch of (Layer 1 and Layer 2) pairs, the fusion segmentation (Fus-Seg) module uses segmentation (Seg) blocks to generate the prediction point labels vector (pre-labels) for each point input. The pre-labels vectors of the same point in the input point cloud are represented as $\left\{F_{1}^{\left(n\right)},\;F_{2}^{\left(n\right)},\;F_{3}^{\left(n\right)},\;\cdots ,\;F_{C}^{\left(n\right)}\right\}$ and $\left\{S_{1}^{\left(n\right)},\;S_{2}^{\left(n\right)},\;S_{3}^{\left(n\right)},\;\cdots ,\;S_{C}^{\left(n\right)}\right\}$. The segmentation (Seg) block is a learnable operation that is trained by finding the best across pairing in the multi-embedding space [32]. To enhance the interactivity between feature extraction layers in the network, the Fus-Seg module introduces a multi-embedding space by two jointly input layers’ pre-labels, which ensures that all pairings that cross two pre-labels actually occur in the space. The advantage of the multi-embedding space is that it can maximize the cosine similarity of real pairings in the point cloud, and minimize the cosine similarity of incorrect pairings. The size of the multi-embedding space is C×C, and C depends on the number of segmentation parts when the network is training. The across pairings in the multi-embedding space are represented as $F_{i}^{\left(n\right)}\cdot S_{j}^{\left(n\right)}$. When i=j, the across pairings are seen as real pairings, and when i≠j, they are incorrect pairings.

In addition to increasing the communication of the feature extraction layers in the network, the Fus-Seg module tags points to enrich the features of point clouds. After the multi-embedding space, max pooling is adopted to obtain a label vector for each point. The size of a label vector is 1×C, and after max pooling, the size of the label matrix of the point cloud is N×C. Then, the label matrix is fed back to per point features by matrix multiplication, with the features of Layer 2 with each of the point features. The label matrix is regarded as an annotation, which can be used to extend the dimension of the feature map, which is (N×f→N×f×C). Moreover, the label matrix is full of information with segmentation scores, which means the label matrix can be seen as a weight matrix as well. It can be used to sift evaluation factors in the feature maps and enhance the effect of features that play a more important role in the process of network segmentation.

### 2.3. DFSNet Structure Design

The DFSNet structure is composed of an input layer, a local feature aggregation module, the dynamic segmentation layer, and the output layer. The details of each part are as follows:

Input layer: The input data of the network constitute the point clouds of the orchard scene, which are collective in nature. Each point in point clouds has three column vectors (X, Y, Z), which represent the 3D coordinates. From the perspective of the input data structure, the size of the input point is N×f, where N is the number of point clouds and f is the feature dimension of each data point. Moreover, considering that the point clouds collected by the LiDAR equipped on the plant protection robot are relatively sparse, the point clouds need to be down-sampled before entering the training phase.

Local feature aggregation (LFA) layer: The LFA module consists of position encoding and 3 stages of SA layers. These SA layers are used to aggregate the features of the point cloud and increase the feature size of each point of each point cloud. In the data of the SA layer, the data size of the point cloud data is reduced to 1/2 of the original data size after each stage. In the network, the input point cloud number is N. After three data SA layers, the size of the point is (N→0.5N→0.25N→0.125N). Then, a reconstruction operation that is performed on the point cloud makes the number of point clouds back to N. Meanwhile, the feature of the point size is gradually up, and the dimension of the local feature is f=2160.

The dynamic segmentation layer: The dynamic segmentation layer includes a Fus-Seg module and an inverted residual block [33]. The Fus-Seg module is used in the network to connect the layers and enhance the expression ability of each point. After the Fus-Seg module, the change in the feature map of the point cloud is (N×f→N×f×C). The inverted residual block that includes three convolutions is used to learn features of the point cloud. The inversion operation allows significant reduction in the memory footprint needed during inference by never fully materializing large intermediate tensors. The block takes as an input a low dimensional compressed representation, which is first expanded to a high dimensional representation; then, the feature is subsequently projected back to a low dimensional representation, that is, (f→2f→f).

Output layer: The output layer of the network is a fully connected layer, the function of which is to map the learned features to the sample label space. The output of the network is the predicted semantics of all points with a size of N×C, where C is the number of classes in the dataset. In the simple-scale dataset, C=5. In the complex-scale dataset, C=8.

**Figure 3:**
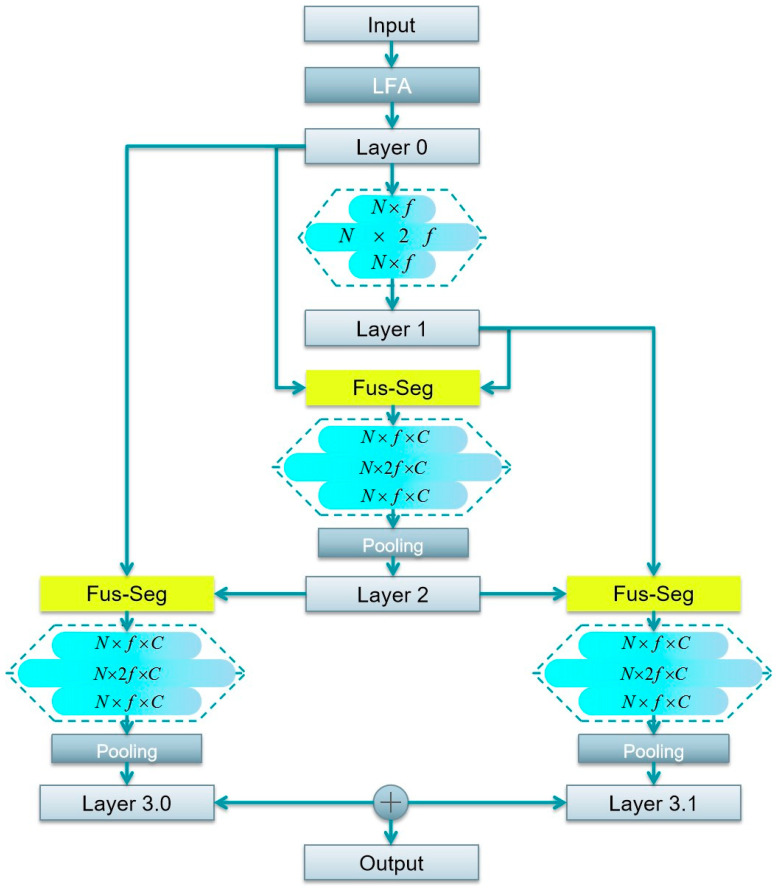
The structure of DFSNet. LFA, local feature aggregation layer; Fus-Seg, fusion segmentation modules; MLP, shared multi-layer perceptron.

The specific DFSNet architecture is shown in Figure 3 below.

## 3. Results

### 3.1. Dataset Details

**Figure 4:**
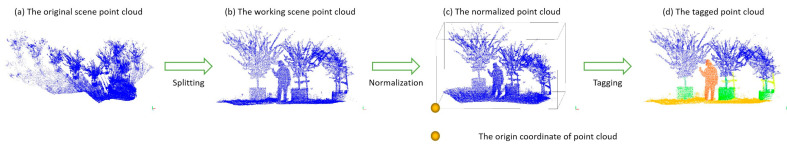
The visualization of point cloud data processing. (**a**) The original scene point cloud, which is filtered from the collected point cloud. (**b**) The working scene point cloud, which is split from the original scene point cloud. (**c**) Normalize the coordinates of each set of point clouds. (**d**) Segment different parts of the point clouds of the scene and represent them in different colors for labels of the segmentation results.

In this study, we used a self-made dataset collected in a real nursery scene. The dataset satisfies the requirements of unstructured forestry scenes, and can be used to measure the performance of the semantic segmentation network. The experimental site selected for this study is a nursery located on 32.12° N. 119.31° E, Zhenjiang, Jiangsu, China. The nursery provides varieties of common landscape orchards and forestry scenes to make the trained models of deep learning networks obtain different application scenes. To ensure sufficient growing space, a distance of 2 to 5 m is maintained between each tree trunk. Moreover, all of the trees are planted in cultivation pots, and some of the trunks are supported by sturdy wooden stakes. To create experimental datasets, we utilized the Livox Horizon scanner to collect the original point clouds. And the CloudCompare (Paris, France) played an important role on subsequent processing of collected point clouds. The software is employed to filter the noise point clouds, split the original scene point cloud after filtering into smaller working scenes, normalize these point clouds of working scenes, and tag segmented point clouds. Figure 4a to Figure 4b show the processing of a splitting of an original scene into a working scene. A typical complete point cloud for a working scene (2–3 trees and other objects in the orchard scene) normally includes 10–60 k points after split processing. For better trained models of network generalization, working scene point clouds need to be normalized on coordinate positions. Considering the perspective of LiDAR when the network is deployed to the orchard robot, we framed the point cloud of working scenes, and set the lower left point of the box as the origin coordinate, then calculated the coordinates of points in the point cloud by translation. An example of a group of normalized point clouds is depicted in Figure 4c. Then, we tagged the point, and the results of a group of scenes are shown in Figure 4d. The format of the dataset files is .txt, and the files include 3D coordinates of point clouds and labels of each point shown as (X, Y, Z, Label).

**Figure 5:**
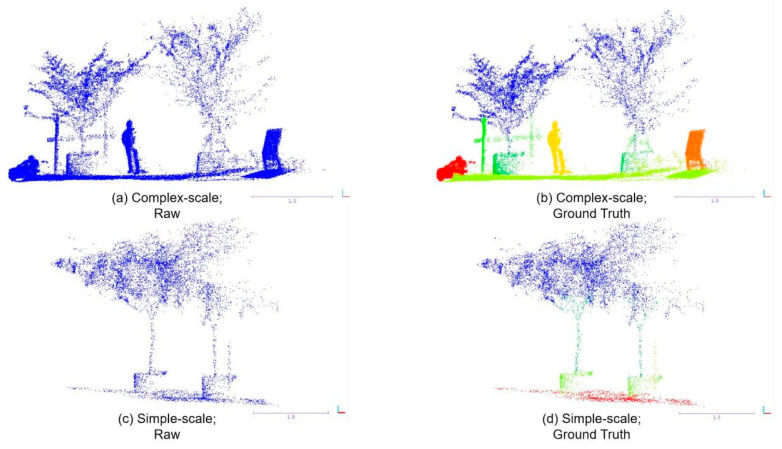
The raw point clouds and their segmentation results; different colors represent different labels of segmentation. (**a**,**b**) represent the complex-scale segmentation. (**c**,**d**) represent the simple-scale segmentation.

To obtain a greater quality of point cloud data for network training based on the distinction of work scenes complexity, we further divided the segmentation into two cases: a simple-scale dataset and a complex-scale dataset. The simple-scale dataset contains only trees and grounds. The complex-scale dataset stands for orchard scenes containing more objects, such as people, indicators, and other objects. Examples of the raw and labeled point cloud and semantic segmentation results of the scenes are shown in Figure 5. To realize the 3D point cloud semantic segmentation of the natural orchard scene, the complex-scale dataset and simple-scale dataset are used as the training dataset and test dataset. In the complex-scale dataset, eight semantic labels were applied for the training set, namely, leaves of trees (leaves), trunks of tree (trunks), pots for planting trees (pots), scaffolds for protecting the growth of trees (scaffolds), grounds, people, indicators, and other objects (others). In the program, the corresponding values were set for these eight tags, i.e., 0, 1, 2, 3, 4, 5, 6, and 7. In the simple-scale dataset, five semantic labels were applied for the training set, namely leaves, trunks, pots, scaffolds, and grounds. The corresponding values were set for five tags, i.e., 0, 1, 2, 3, 4, and 5. The trained network can be used to segment tree phenotypes in orchard environments. In addition, the trained model can also detect some obstacles in the orchards. The model plays an important role in the perception system of agricultural robots, and can be used in the autonomous control system and the autonomous navigation of plant protection robots. The complex-scale dataset used 206 of 309 scenes to create the dataset for training, and the rest of the scenes were used to perform training validation and evaluation. And the simple-scale dataset used 360 of 535 scenes to create the dataset for training. Each dataset includes from 30,000 to 60,000 points, and 4096 randomly sampled points in every point cloud as pre-processing before input to the network.

### 3.2. Experimental Hardware Equipment

The network was developed based on PyTorch-1.13.1. The network training and testing were performed using an NVIDIA RTX-3080Ti (12 GB).

### 3.3. Training Details

**Figure 6:**
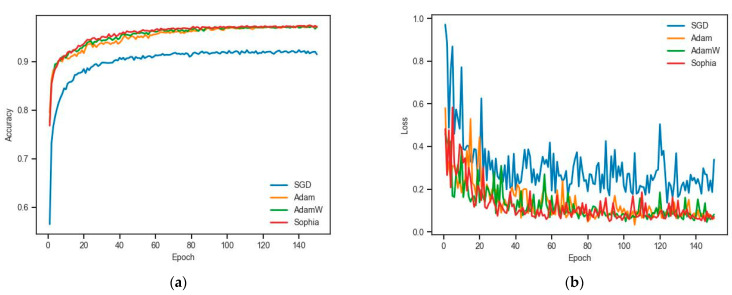
(**a**) The training accuracies and training epochs and (**b**) the loss functions and training epochs.

When training a deep learning network, a faster optimizer can improve the efficiency of network training, which reduces the time cost of implementing the same network training, or achieves a smaller error under the same budget. We mainly compared SGD, Adam [34], and AdamW [35] with Sophia [36], which are dominantly used optimizers on deep learning networks. The hyper parameters of optimizers during the network training are shown in Table 2. Figure 6 illustrates the training accuracy curve and loss curve on DFSNet with the same number of hyper parameters. Sophia achieves better accuracy and better stability loss than SGD, Adam, and AdamW. Thus, we used Sophia as the network optimizer during training.

### 3.4. Semantic Segmentation on Benchmarks

In the field of 3D point cloud semantic segmentation, the mIoU and Acc are the two main indicators used to evaluate the effect of semantic segmentation. The mIoU is an important indicator for measuring the accuracy of the segmentation. The IoU mainly calculates the ratio between the intersection and union of the two sets. The mIoU calculates the IoU according to each class, and then takes the average.

(3) $$mIoU=\frac{1}{C+1}\sum_{i=0}^{C}\frac{S_{ii}}{\sum_{j=0}^{C}S_{ij}+\sum_{j=0}^{C}S_{ji}-S_{ii}}$$

$S_{ii}$ is the real quantity, and C+1 is the number of classes (including empty classes). $S_{ij}$ represents the number of predictions for the true value of i as j, and $S_{ji}$ represents the number of predictions for the true value of j as i. $S_{ij}$ and $S_{ji}$ represent false positives and false negatives, respectively.

The Acc is the simplest metric calculation, the probability that the semantic annotation result of each sample will be consistent with the actual data annotation type. The accuracy is the ratio between the model’s correct predictions on all test datasets and the overall number.

(4) $$Acc=\frac{T}{N}$$

T represents the number of the model’s correct predictions on the test datasets, and N represents the overall number of all the test datasets.

### 3.5. Processing of Network Building

We first analyzed the effect of the number of network layers on segmentation performance. Table 3 shows the training results for the different number layers of the network. The output layer means the number of the dynamic segmentation layer of the network. For example, if the output layer is three, there are three dynamic segmentation layers. When the number of the layer is three, the Acc and mIoU reached their optimal values of this training; the optimal segmentation accuracy is 78.89%, and the mIoU is 73.02%.

**Figure 7:**
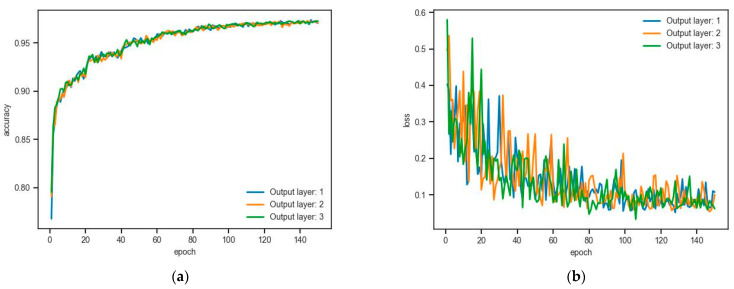
Function graphs of the relationships between (**a**) the training accuracy and the training epoch and (**b**) the loss function and training epoch.

Figure 7 is the training result of the tensorboard. Figure 6a shows the relationship between the segmentation accuracy (Acc) and the number of iterations (epoch), and Figure 6b shows the function graph of the relationship between the loss function and the number of iterations (epoch).

It can be seen from the above training log that when the number of epochs is less than 20, the segmentation accuracy already reaches 0.8, and when the number of epochs = 60, the segmentation accuracy reaches 0.95. In addition, the loss function continues to decrease with a continuous increase in training times, and finally remains at approximately 0.05. Compared to the other numbers of layers, the three-layer network is smoother, proving it is steadier.

### 3.6. Sampling and Grouping Strategy

**Figure 8:**
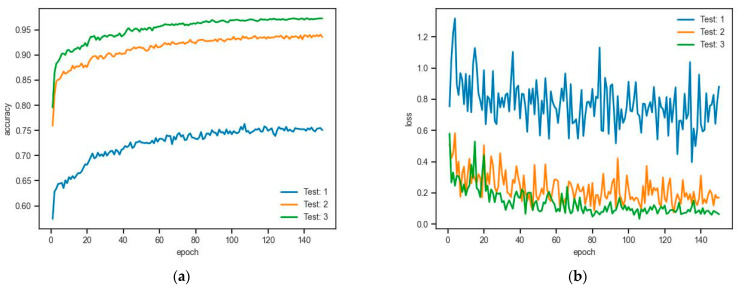
Function graphs of the relationships between (**a**) the training accuracy and the training epochs and (**b**) the loss function and training epochs.

DFSNet applies an iterative farthest point sampling (FPS) or random sampling (RS) strategy to sample (N→0.5N→0.25N→0.125N) centroids from a point cloud with N points, and then uses k-nearest neighbor (k-NN) to gather k=64 points within the neighborhood of each centroid. Table 4 shows the evaluation of segmentation accuracy on centroids sampling and neighbor points grouping strategy by using the complex-scale dataset (N=4096) on the three-layer DFSNet. Simultaneously, a line graph is plotted in Figure 8 to illustrate the training results of the ablation study in the three strategies, demonstrating the superiority of the sampling layer with FPS and k-NN. Test 1 is the model that only uses the FPS sampling layer without the grouping strategy. Experimental results show that grouping strategy is necessary, though the network iterates faster without it. Test 2 and Test 3 compare the sample layer that uses FPS and RS sampling strategies, respectively. The experimental results show that the sample layer with FPS achieves a higher score compared to the layer using RS.

**Figure 9:**
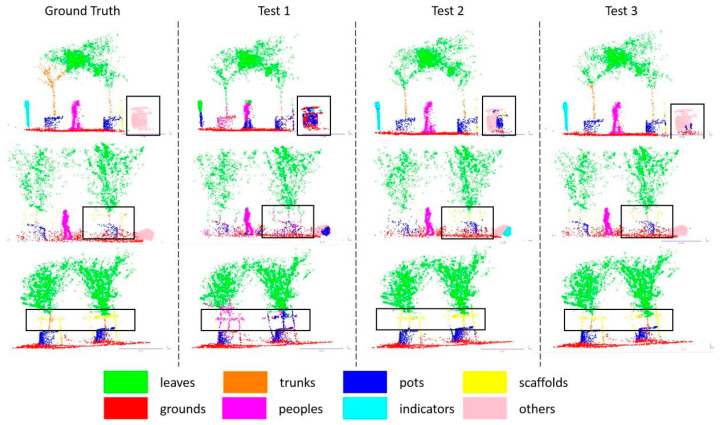
Complete semantic segmentation using the different sampling strategies. Different colors correspond to different segmentation labels. The black boxes mark the segmentation error parts in the point cloud.

Figure 9 shows the results of the semantic segmentation of the 3D point cloud obtained by these three sampling strategies on the simple-scale and complex-scale datasets. The experiment results show that the semantic tags that the network can segment include leaves of trees, trunks of trees, trees planted in pots, scaffolds, grounds, people, indicators, and others. In the orchard scenes, Test 3 shows the best semantic segmentation effect during these sampling and grouping strategies, and Test 3 has the strongest generalization ability. From the comparison results of the segmentation in black boxes in the figure, Test 3 can clearly segment indicators, people, others, and some details in the orchard field.

### 3.7. Segmentation in Orchards

**Figure 10:**
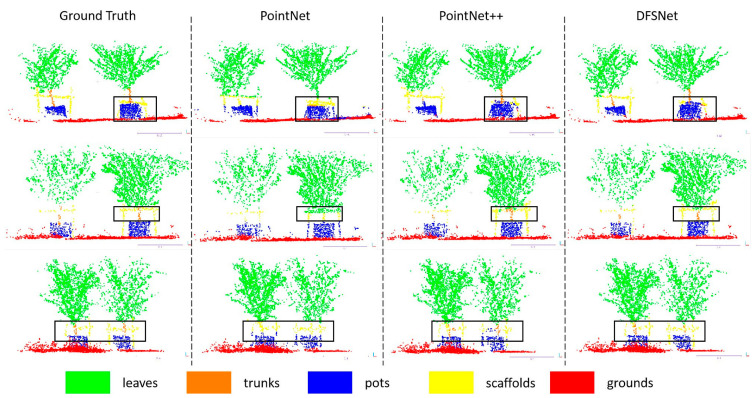
Semantic segmentation results of different networks in the simple-scale dataset. The black boxes mark the segmentation error parts in the point cloud.

**Figure 11:**
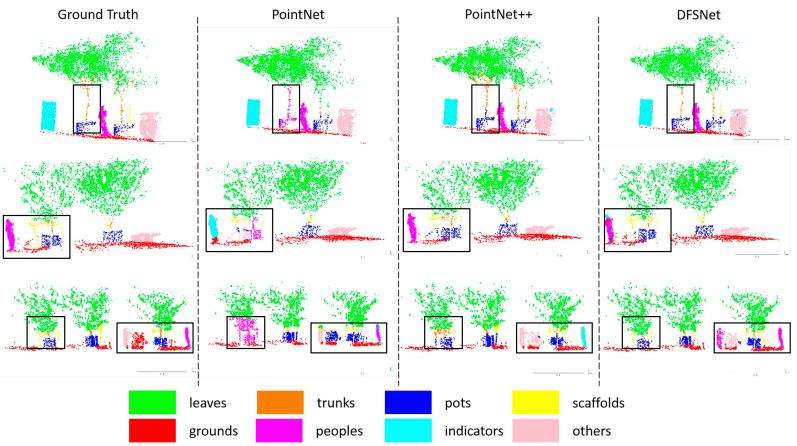
Semantic segmentation results of different networks in the complex-scale dataset. The black boxes mark the segmentation error parts in the point cloud.

This section evaluates the network performance in the simple-scale and complex-scale datasets, and compares it with PointNet, PointNet++, and Point-NN. We first trained the simple-scale and all-scale dataset (combines the simple-scale dataset and the complex-scale dataset) separately by these networks, then tested the networks on different datasets via trained models. According to the quantitative results in Table 5, DFSNet outperforms these typical networks in orchard fields in the segmentation metrics. Among them, when networks both train and test on the simple-scale dataset, the accuracy of DFSNet improves performance by 5.6% over PointNet, and the mIoU improves by 22.32% over PointNet, 1.62% over PointNet++, 1.14% over D-PointNet++, 7.28% over DGCNN, and 25.46% over Point-NN. When the all-scale dataset is used as the training dataset and networks were tested on the simple-scale dataset, we see an 8.19% improvement over PointNet, a 1.12% improvement over PointNet++, a 0.81% improvement over D-PointNet++, and a 1.39% improvement over DGCNN in Acc; then, we also see a 36.58% improvement over PointNet, a 9.27% improvement over PointNet++, a 7.78% improvement over D-PointNet++, a 9.06% improvement over DGCNN, and a 22.12% improvement over Point-NN in mIoU. While testing networks on the complex-scale dataset, the proposed network improves orchard fields segmentation accuracy by 14.14% over PointNet, by 4.96% over PointNet++, by 4.10% over D-PointNet++, and by 3.91% over DGCNN. The network improves mIoU by 22.77% over PointNet, 8.41% over PointNet++, 3.44% over D-PointNet++, 8.49% over DGCNN, and 25.48% over Point-NN. Overall, using networks to segment the scenes on the all-scale dataset, we obtain a 11.73% improvement in Acc and a 28.19% improvement in mIoU when compared to PointNet, a 3.76% improvement in Acc and a 9.89% improvement in mIoU compared to PointNet++, a 2.36% improvement in Acc and a 6.33% improvement in mIoU when compared to D-PointNet++, a 2.74% improvement in Acc and a 9.89% improvement in mIoU over DGCNN, and a 24.69% improvement in mIoU over Point-NN. Figure 10 and Figure 11 show the visualization of the segmentation. As shown in the figures, our DFSNet can accurately segment tree point clouds and recognize objects from the orchard to achieve on par or better segmentation results when compared to PointNet and PointNet++. PointNet produces many false negatives for the points, which is due to the global features without aggregation. PointNet++ has apparent benefits, as compared to PointNet, for segmenting the scenes, but it is still experiences interference by the coordinate values of points, and does not achieve precise edging of different categories. During the experiments, the ability of DFSNet is significantly the best over PointNet, PointNet++, and Point-NN. The improvement in the proposed network may be attributed to the pre-processing local feature aggregation layer and the dynamic segmentation layer, and its ability to better extract point cloud features of orchard environments through DFSNet learning.

## 4. Conclusions

Orchards play a crucial role in agricultural production. To facilitate the efficient management of orchards and the practical applications of agricultural robots, a sensory perceptual system is necessary. In this research, a 3D point cloud semantic segmentation network called DFSNet for the unstructured orchard fields was proposed, and some good semantic segmentation results were achieved. The proposed network utilized a local feature aggregation (LFA) module and three fusion segmentation (Fus-Seg) modules to improve the training performance when dealing with imbalanced class problems. Meanwhile, the impacts of network depth and 3D point cloud sampling and grouping strategies on the semantic segmentation were compared and analyzed. The test results showed that deeper deep learning neural network is helpful for improving the accuracy of semantic segmentation, and the best sampling strategies we studied use FPS to sample and k-NN as the grouping strategy. The key to semantic segmentation depends on whether the relevant features in the 3D point cloud data can effectively be extracted. The training experimental results show that the best accuracy of 3D point cloud semantic segmentation can reach 89.43%, and the mIoU can reach 74.05% on the datasets that combine the simple-scale dataset and the complex-scale dataset. Comparing DFSNet with the other networks, the accuracy value of the proposed network is 11.73%, 3.76%, 2.36%, and 2.74% higher than the PointNet, PointNet++, D-PointNet++ and DGCNN, respectively. Meanwhile, the mIoU of the proposed network is better by 28.19%, 9.89%, 6.33%, 9.89%, and 24.69% compared with the PointNet, PointNet++, D-PointNet++, DGCNN, and Point-NN, respectively. Both the accuracy and mIoU ensure quality segmentation of the orchard scene.

In practical applications, the proposed DFSNet can provide more accurate information about the orchard scenes such as ground filtering, object identification, tree phenotyping, and other related information. Ground and object detection is the basis for accurate identification of tree row paths, with path planning being the key to orchard management. Additionally, tree phenotype information such as crowns and trunks can be used to realize target locations, which is beneficial to agricultural robots tasks, for example, autonomous variable rate spraying, autonomous fruit picking, and so on. Thus, the proposed network holds practical significance for orchard management. In subsequent studies, we intend to obtain more experimental results via our hardware platform and perception algorithm designed for orchard environments.

## Figures and Tables

**Table 1:** Systematic comparison among some representative methods.

Network	Sampling + Grouping Strategy	Principal Operator	Basic Architecture
PointNet	-	MLP ^3^	Branch
DGCNN	k-NN ^1^	EdgeConv	Branch and Dynamic
PointMLP	Geometric affine module	MLP	ResNet
PointNet++	FPS ^2^ + Region grouping	PointNet layer	U-Net and Hierarchical
D-PointNet++	FPS + Region grouping	Dense PointNet layer	DenseNet and PointNet++
HorNet	-	g^n^Conv ^4^	HorBlock and FFN ^5^

^1^ k-NN: k-nearest neighbor; ^2^ FPS: farthest point sampling; ^3^ MLP: multi-layer perceptron; ^4^ g^n^Conv: recursive gated convolution; ^5^ FFN: feed-forward network.

**Table 2:** Hyper parameters of optimizers during the DFSNet training.

Hyper Parameters	Value
lr ^1^	0.001
betas	(0.9, 0.999)
epc ^2^	1 × 10^−8^
weight_decay	1 × 10^−4^
Epoch	150

^1^ lr: learning rate; ^2^ epc: error of floating-point calculation.

**Table 3:** Comparison of layers on network performance.

Output Layer	Acc (%)	mIoU (%)	Iterate Speed (ms/pc *)
1	72.16	62.28	13.79 ms
2	72.18	63.13	13.88 ms
3	78.89	73.02	14.08 ms

* pc: point cloud.

**Table 4:** Comparison of sampling and grouping strategies on network performance.

Test	Sampling + Grouping Strategy	Acc(%)	mIoU(%)	Iterate Speed
1	FPS ^1^	37.20	32.17	4.76 ms
2	RS ^2^ + k-NN ^3^	75.64	66.70	17.38 ms
3	FPS + k-NN	76.57	69.16	13.98 ms

^1^ FPS: farthest point sampling; ^2^ RS: random sampling; ^3^ k-NN: k-nearest neighbor.

**Table 5:** Results on semantic segmentation in orchard fields.

Train Dataset	Test Dataset	Network	Acc (%)	mIoU (%)
Simple-scale	Simple-scale	PointNet	92.02	63.08
		PointNet++	97.24	83.78
		D-PointNet++	97.27	84.26
		DGCNN	95.96	78.12
		Point-NN	-	59.94
		Ours	97.62	85.40
All-scale (Simple-scale + Complex-scale)	Simple-scale	PointNet	89.03	49.71
		PointNet++	96.10	77.02
		D-PointNet++	96.41	78.51
		DGCNN	95.83	77.23
		Point-NN	-	64.17
		Ours	97.22	86.29
	Complex-scale	PointNet	71.69	43.95
		PointNet++	80.87	58.31
		D-PointNet++	81.73	63.28
		DGCNN	81.92	58.23
		Point-NN	-	41.24
		Ours	85.83	66.72
	All-scale (Simple-scale + Complex-scale)	PointNet	77.70	45.86
		PointNet++	85.67	64.16
		D-PointNet++	87.07	67.72
		DGCNN	86.69	64.16
		Point-NN	-	49.36
		Ours	89.43	74.05

## Data Availability

Data will be made available upon request.
